# Retraction Note to: The lncRNA XIST promotes colorectal cancer cell growth through regulating the miR-497-5p/FOXK1 axis

**DOI:** 10.1186/s12935-021-02205-2

**Published:** 2021-09-14

**Authors:** Nan Wang, Jia‑Xing He, Guo‑Zhan Jia, Ke Wang, Shuai Zhou, Tao Wu, Xian‑Li He

**Affiliations:** Department of General Surgery, Tangdu Hospital, The Air Force Medical University, Xi’an, 710038 China

## Retraction to: Cancer Cell Int (2020) 20:553 10.1186/s12935-020-01647-4

The Editors-in-Chief have retracted this article. Concerns were raised regarding Figure 2, specifically:

- Figure 2c: part of the top right panel appears to be partly covered by a white circle.
- Figure 2d: the si-NC panels appear to partially overlap.
- Figure 2e: the si-XIST HT29 panel appear to partially overlap with the siXIST HT29 panel and the si-XIST SW480 panel of Figure 2d.

The Editors-in-Chief therefore no longer have confidence in the reliability of the data reported in the article.

Xian-Li He has stated on behalf of all authors that the authors agree with this retraction.

